# Entrepreneurs' Well-Being: A Bibliometric Review

**DOI:** 10.3389/fpsyg.2018.01696

**Published:** 2018-09-12

**Authors:** José Carlos Sánchez-García, Gioconda Vargas-Morúa, Brizeida Raquel Hernández-Sánchez

**Affiliations:** Universidad de Salamanca, Salamanca, Spain

**Keywords:** well-being, entrepreneur, bibliometric review, social entrepreneur, self-employment, business owner, independent worker, organizational employer

## Abstract

The present article aims to summarize and classify existing research entrepreneurs' well-being through a bibliometric literature review. Its main objectives are: to identify the different theoretical perspectives and research strands that characterize and define literature on entrepreneurs' well-being and highlight the connections between them; as well to look for emerging trends and gaps in its literature by comparing the most recent works with those that represent the field's core. The document is based on bibliometric data: it uses citation techniques to select, analyze, and interpret citation patterns within the literature on entrepreneurs' well-being. The paper identifies six main groups, as well as several specific research flows and common themes that represent academic publications on entrepreneurs' well-being. The research strands on the topic are grouped into six different theoretical perspectives grounded in entrepreneurship related to: culture, education, innovation, sustainable development and small business; psychological well-being; social entrepreneurship and economic development; women and employment; and self-employment; life satisfaction and economic growth, and business administration. Data from the most recent publications were used to verify whether original topics and themes are reflected in contemporary debate and in which fashion. Limitations related to search engines, such as missing keywords were accounted by utilizing three different database as well as expanding keyword number. From a practical perspective, this research is expected to contribute on theory construction, management decision making, and teaching. This study describes the growing development of the literature on entrepreneurs' well-being, and the underlying structure of the different streams of research therein.

## Introduction

In the last decade, the entrepreneurial activity has shown a growing development that affects governments, societies and people in general and, with the same impetus, this has been reflected in entrepreneurship research.

According to the Global Entrepreneurship Monitor (2017–2018) report, entrepreneurship levels are stable or increasing worldwide. Seventy-four percent of respondents said they had chosen to seek for opportunities as basis for their business motivations, and 43% of the world's population sees good opportunities to start a business in the next 6 months. In addition, almost 70% of the adult population across 52 economies around the world believe that entrepreneurs are well appreciated and enjoy high status within their societies.

Likewise, this growth is reflected in the increase of academic papers related to the field of entrepreneurship (Web of Science, 2017) in developed countries in developed countries (Bruton et al., 2008) such as the United States, England (Trueman et al., 2013), and Germany (Hetschko, 2016), with issues related to the well-being of the entrepreneur, such as the work-family conflict (Nguyen and Sawang, 2016), self-employment (Binder and Coad, 2013), stress (Cardon and Patel, 2015), firm performance (Hmieleski et al., 2013), innovation (Baron and Tang, 2011), creation of financial, social and environmental wealth (Zahra et al., 2014), as well as the possible implications depending on the type of entrepreneurship (Uy et al., 2017) and on the suitability of entrepreneurs and businessman when running their companies (Stock et al., 2016).

However, not all studies define the concept “entrepreneur,” instead they presuppose an interpretation and, on many occasions, their definition is conditioned by the context of research. Initially Knight (1964) provides some characteristics of an entrepreneur and indicates that it will be the person who takes risks and occupies a position of uncertainty proposed, one who takes the initiative, who has imagination and creates new opportunities, that is, “…a state of mind to direct personal attention, experience and action toward a specific goal or access to achieve something” (Bird, 1988, p. 442).

Lumpkin and Dess (1996) consider that the entrepreneurial orientation refers to the processes, practices and decision-making activities, and actions that work in a dynamic generator process aimed at venture creation. Its key feature corresponds to a propensity to act autonomously, willingness to innovate and take risks, as well as a tendency to be aggressive toward competitors and proactive with regard to market opportunities.

Repeatedly, is conceived a person with entrepreneurial orientation as one that combines innovation, takes risks and is proactive (Miller and Friesen, 1983; Hansen et al., 2011; Goktan and Gupta, 2013) and “entrepreneurship” is understood as the ability of people to translate ideas into actions. It means to be creative, lead, innovate, take risks, and manage personal and professional projects to achieve specific objectives (Sánchez, 2013; Oliver et al., 2015).

Finally, Shane y Venkataraman provides a definition of “entrepreneurship,” as a “scholarly examination of how, by whom, and with what effects opportunities to create future goods and services are discovered, evaluated and exploited” (2000, p. 218).

There is no doubt that entrepreneurship is a challenging effort, which entails great challenges for an entrepreneur. As we have seen in the definitions, it involves taking risks, making decisions, taking advantage of opportunities, acting in uncertain environments in a proactive and innovative way, in order to achieve objectives that are specific to each entrepreneur. This leads us to reflect whether in the midst of all this process, entrepreneurs actually manage to reach the condition of well-being.

It is worth mentioning that the term well-being was used even before Aristotle (IV century BC) and the issue has not gone unnoticed since then, but it is from this last decade that there has been an important increase in its study, in search of greater understanding of its link with entrepreneurship (Uy et al., 2013).

Well-being has gone from the popular perception of “being well” to being considered a measure of global interest that must be included in the analysis of human development. Such is the importance of this topic that has recently been incorporated into the official statistics of different countries (INEGI, 2013) since it is considered that the follow-up of this allows to make better governmental, business and social decisions. It has also been addressed in global forums (OCDE, 2011), in a large number of organizations (Stiglitz et al., 2009; INEGI, 2013; Survey World Values., 2017) and in programs such as the United Nations Development Program.

Many philosophers have been reflecting on happiness as the origin of well-being, which is intrinsic to human beings. However, after the Second World War, studies related to the people's well-being were associated with the treatment of the disease their causes. It is only after the emergence of positive psychology (Seligman, 1998, 2004) that well-being research, and its traditional conception associated with negative and pathological aspects of the human being, takes a turn to give way to the foundations of psychological well-being, happiness, strengths and human virtues.

Research on well-being has been approached from different perspectives, such as sociological (Veenhoven, 2008); economic (Clark and Oswald, 1996; Deci et al., 2001; Easterlin, 2001; Van Praag et al., 2003; Brown et al., 2008); and psychological (Diener et al., 1999; Park et al., 2004; Kahneman et al., 2006; Forgeard et al., 2011; Forgeard and Seligman, 2012; Csikszentmihalyi and Larson, 2014). Therefore, efforts are now focused on studies that support the construct of “well-being” as a combination of feeling good and having purpose and meaning in life, good relationships, family support; have a rewarding, attractive job, adequate income; be reasonably healthy, have important goals related to personal values; live in a democratic environment and a stable society (Diener and Seligman, 2004).

Well-being can be explained in many ways, and is generally associated with the measurement instruments used (DeNeve and Cooper, 1998). However, we will define it in hedonic and eudaimonic perspective for better comprehension.

Hedonic well-being refers to happiness in terms of achieving pleasure and avoiding pain (Deci and Ryan, 2000). The hedonic point of view focuses on subjective well-being, which is defined as the presence of positive affect and greater satisfaction with life, as well as the absence of negative affect (Diener, 1984). That being the case, we understand satisfaction with life as a cognitive, judicious process, that is, a global assessment of a person's quality of life according to their chosen criteria (Shin and Johnson, 1978). Satisfaction judgments depend on a comparison of circumstances with what is believed to be an appropriate standard, as such, it does not depend on criteria that the researcher deems important or imposed externally, but on those that people establish for themselves (Diener, 1984).

The eudaimonic approach focuses on meaning and self-realization and defines well-being in terms of the degree to which a person is fully functioning (Ryan and Deci, 2001). It is defined as a fully functional and self-realized individual (Deci and Ryan, 2000). Vigor, also called vitality, is a common operationalization of eudaimonic well-being (Deci and Ryan, 2000; Ryan and Deci, 2001). The concept of subjective vitality refers to the state of feeling alive and alert, to having available energy, that is to say of positive physical functioning, for what is considered an aspect of eudaimonic well-being (Ryan and Frederick, 1997), since it is vital and energetic it is part of what it means to be in full psychological functioning.

Taking into account the previous points, we recognize that existing research in entrepreneurship provides a solid foundation for further development and, through it, can determine those aspects that have been mostly addressed to achieve entrepreneurial well-being. However, all the information related to the topic is presently not structured, and thus, not possible to understand from an overview.

To try to correct this gap in the research field, this article provides a bibliometric information platform that shows the research areas that have been studied, authors, sources and years of development, which encompass subjective well-being on entrepreneurs. For the literature search, we used the Thompson Reuters' Social Science Citation Index (SSCI), Elsevier's Scopus and ProQuest databases with the following specific objectives in mind:

1. Identify the different theoretical perspectives and research streams that characterize and define literature on entrepreneurs' well-being, and highlight the connections between them.
2. Identify emerging trends and gaps in the literature by comparing the topics considered.

We ensure that the quality of the information was excellent and could respond to the research questions: in which areas of study the entrepreneurs' well-being becomes relevant?, what has been its importance over time?, what are its major research proponents?, and what are its mayor journal sources?

In this sense, this article integrates the information found and groups it according to the different perspectives that deal with the subject of entrepreneurs' well-being.

## Methods

### Study design

In this article, we carry out a bibliometric review of the literature on entrepreneur's well-being, which seeks to synthesize this scientific literature. We have used strict control mechanisms in order to reduce biases to a minimum, such as the PRISMA method (Liberati et al., 2009; Urrútia and Bonfill, 2010) in the process of choosing and discarding articles. In addition, we have relied on a previous protocol of explicit criteria, uniformly applied to all articles, in order to narrow the topic and focus on the objectives set.

Only peer-reviewed articles have been used in order to guarantee the reduction of bias as much as possible and, in turn, this allow us to determine the frequency and relationship of the most co-cited authors of the topic; the frequency and relation of the sources in which it is mostly published on, and the progress in its research through time.

This bibliometric study includes the following steps: 1. Selection of articles related to entrepreneurial wellbeing 2. Application of statistical methods to extract relevant information and 3. Inclusion of a narrative synthesis about the major findings of the study.

Our structure of analysis of bibliometric networks is based on an approach for unified mapping and grouping (van Eck and Waltman, 2010; Waltman et al., 2010), which provides information on the structure of a network, on the fields of research, how they relate to topics among themselves and how the subject has developed over time. As such, through this quantitative and qualitative analysis of the evidence found, we are able to answer our research questions.

The search for relevant articles, procedures, and reviews we used the Social Science Citation Index (SSCI), Scopus and ProQuest database, without defining a certain time frame of publication. These were chosen because the science portion of SSCI covers all of the major English-language international journals (Goodman and Deis, 2005), while Scopus offers sorting and refining features so that researchers can easily find and access more than 27 million abstracts and citations stretching back to the mid-1960s (Boyle and Sherman, 2006) and ProQuest as a third and complementary source, as it offers a wide repository of business and social science research.

### Search strategy

Citation techniques and the PRIMA method (Liberati et al., 2009; Urrútia and Bonfill, 2010) were used to select, analyze, and interpret citation patterns within the entrepreneurs' well-being literature in two stages. Once the reviewer approved the protocol, we proceeded to look for the information.

In three databases (SSCI, Scopus and ProQuest), we used the systematic procedure outlined below:

We used the search terms “well-being” and “entrepreneur^*^.” For the first term, it was searched with and without a space between the two words (wellbeing and well-being) and included quotation marks so that the search engine would identify both words together, and for the second term, we included the asterisk so that the search engine would find all its possible variations.

We understand that many investigations related to the traits and actions of entrepreneurs, such as “self employment,” there is “business owner,” “independent worker,” and “organizational employer” as well. These words were included in our search; however, for purposes of maintaining quality in our study, we only considered peer-reviewed articles and that are specifically associated with the concept “Well Being,” as seen in Table 1.

**Table 1:** Flow diagram of the studies.

**Systematic review of articles**				
**Identification:**	**No. of articles identified through database searching**	**SSCI**	**Scopus**	**ProQuest**
	Boolean code/Search field	Topic	Keyword, Title, Abstract	Any field except full text
	“Well-being” and “entrepren^*^”	214	251	20
	“Wellbeing” and “entrepren^*^”	59	74	7
	“Well-being” and “self-employ^*^”	100	115	24
	“Wellbeing” and “self-employ^*^”	17	27	8
	“Well-being” and “selfemploy^*^”	1	1	7
	“Wellbeing” and “selfemploy^*^”	0	0	4
	“Well-being” and “independent worker^*^”	2	2	1
	“Wellbeing” and “independent worker^*^”	0	0	1
	“Well-being” and “independ^*^ worker^*^”	4	3	1
	“Wellbeing” and “independ^*^ worker^*^”	0	0	1
	“Well-being” and “organizational employer^*^”	0	0	0
	“Wellbeing” and “organizational employer^*^”	0	0	0
	**Total: Includes (articles)** + **Excludes (2018)**	**397**	**473**	**74**
**Screening:**	**No. of articles excluded:**			
	Less duplicates in the same database	53	47	32
	Less documents that are not peer-reviewed articles	28	0	26
	Total per database	316	426	16
	Total sum of articles		758	
	Less duplicates between the databases		249	
	Less without direct content (Title, Abstract or Keyword)		136	
**Included:**	**Total of Articles Analized**		**373**	

*Own elaboration following the PRIMA method (Liberati et al., 2009; Urrútia and Bonfill, 2010)*.

The first step identified 399 SSCI documents, 501 Scopus documents and 81 ProQuest documents. The information was extracted without defining a specific publication period, as we sought to analyze trends in its research across time. Subsequently, we filtered any document that was not classified as a peer-reviewed, and compared lists to eliminate duplicate articles. We then reviewed the abstract of each article to verify that all selected articles were, in fact, in line with the research topic. Thus, we excluded 398 duplicate articles and 210 articles whose information was unrelated to entrepreneurs' well-being. In total, with the VOSviewer program (van Eck and Waltman, 2010) and Excel, we analyze 373 articles. In this way, we ensured that the quality of the information was optimal and would enable us to answer our research questions.

### Flow diagram of the studies retrieved for the review

In Table 1, we detail in the identification, systematized article extraction, and Boolean codes that were used and the quantity of articles obtained from each of the relationships. With the purpose of being exhaustive in the search and, given that the SSCI and ProQuest databases do not have the filters we used (title, keywords and abstract), the search was extended to “subject” and “any field except full text,” respectively.

In the section of choice, we indicated the number of excluded articles because they were either duplicated in the same database when performing different searches or were not peer-reviewed. Later, when joining the databases, we eliminated the duplicated articles among them, or other documents that, when making the revising their Title, keyword or abstract, were unrelated to the topic. Finally, we indicate the quantity of articles analyzed.

### Data sources, study stages, and data extraction

We used VOSviewer software version 1.6.7 (van Eck and Waltman, 2010) to construct and visualize bibliometric maps through the technique of similarities, as well as to identify clusters and their reference networks (van Eck and Waltman, 2010; Waltman et al., 2010), EndNote and Mendeley were used as citation management software.

With VOSviewer (van Eck and Waltman, 2010) two types of bibliographic maps, one based on distance and the other can be distinguished in the graphics. “Distance-based maps are maps in which the distance between two items reflects the strength of the relation between the items. A smaller distance generally indicates a stronger relation” (van Eck and Waltman, 2010, p. 525), which facilitates the identification of groups of related items. Those based on graphics, lines are drawn between the elements to indicate their relationships. For this study, mostly distance-based maps were used.

#### Data analysis

For data analysis, there are two strategies. First, which corresponds to the cluster analysis obtained with the VOSviewer program and the second, which corresponds to the Bibliometric analysis of the information. In this way, we were able to study our topic's within the academic field and could perceive the progress in research from 1974 to 2017.

##### Cluster analysis

In the first stage, in order to identify all possible research fields and associated variables we carried out a co-occurrence analysis with a minimum of two occurrences per word, for a total frequency of 196 keywords grouped into 15 clusters.

**Figure 1:**
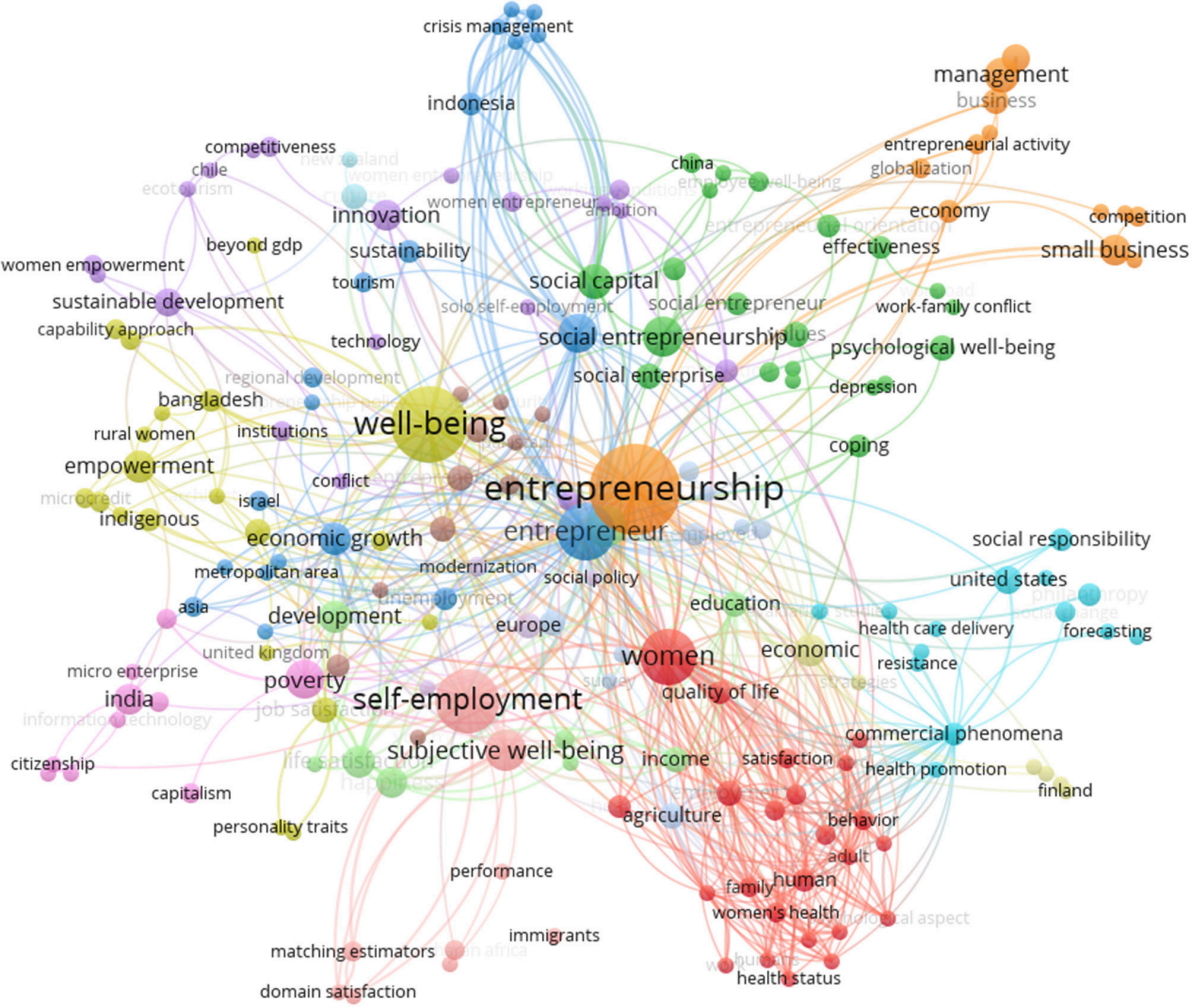
Clusters “well-being” and “entrepreneur^*^.

In this way, the variables were grouped by the system according to criteria of socioeconomic factors and health care policy; female entrepreneur; social entrepreneurship; development and education; self-employment and happiness; small business and management; Agriculture, culture and well-being at work; tourism and entrepreneurship policy; adaptation and social responsibility; economic growth and rural women; economic development and entrepreneurial education; capability approach and innovation; sustainable development; family business; entrepreneurialism; capitalism; citizenship and religion, and relationships. Stronger relations as seen in Figure 1, are represented in circles and with larger labels. For example, entrepreneurship, subjective well-being, self-employment, education, social capital and social entrepreneurship; women and economic growth.

**Figure 2:**
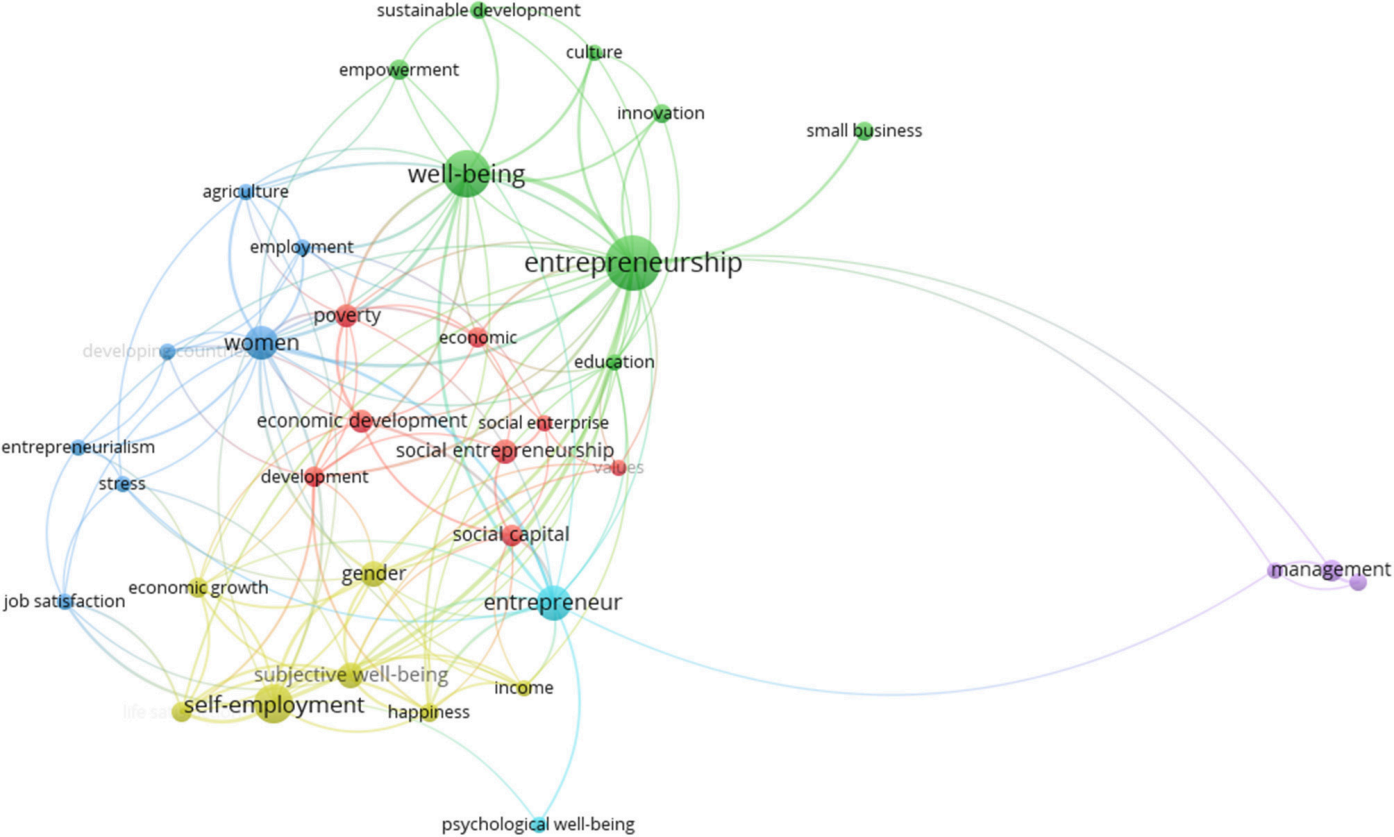
Clusters “Well-being” and “entrepreneurs” with minimum number of occurrences of a keywords:5.

Again, we carried out a co-occurrence analysis with a minimum of five occurrences per word, for a total frequency of 35 keywords with the greatest overall link strength in order to show the six most relevant clusters, as shown in Figure 2.

The combined mapping and grouping shown in Figure 2 provide an overview of the structure in the field of entrepreneurship research. Each group is represented by a different color that exhibits its relative importance, proximity and relationship between them. That is, “the greater the number of neighboring elements and the smaller the distances between these elements and the point of interest, the greater the density of the element” (van Eck and Waltman, 2010, p. 533).

As for the left-side clusters: the first and second have the same amount of items and corresponds to the red and green cluster.

The first cluster in red associates the following keywords: development, economic development, economic, poverty, social capital, social enterprise, social entrepreneurship, and values. The 17.68% occurrence of keywords under study are related to this cluster.

Social entrepreneurship is aimed at developing strategies to support social change and awareness in order to improve the well-being and conditions of people in society. Within this area, we find that the issue has been approached from various perspectives, such as that of social innovation. Dawson and Daniel (2010) indicate that social innovation generates social benefits but can also serve certain commercial, technological, organizational, or scientific purposes, so that the development of social innovation is possible in the organization, the community or society.

Likewise, when considering social innovation the entrepreneur must have a deep knowledge of social problems, and such innovations depend on the active participation of the actors involved and the availability of local endogenous resources (Bernardino and Santos, 2017). The concept of social innovation extends to the field of international entrepreneurship to affect sustainable global well-being, and is a multidimensional concept that includes the creation of financial, social, and environmental wealth (Zahra et al., 2014).

Another aspect that social entrepreneurship must consider is the possibility of contributing to improve the behavioral health problems of entrepreneurs, as well as community, economic, and social development (Ferguson, 2016). Studies have also considered how social initiatives can contribute to local growth and development (Almarri and Meewella, 2015; Bernardino and Santos, 2017), social value, and the integration of well-being (Ferreira et al., 2017).

Also, this cluster it shows a very close relationship to the main terms, because the well-being of the entrepreneur is associated with economic development and social entrepreneurship. For example, to improve the understanding of how micro-level subsistence activities might be related to higher-level phenomena to increase the well-being of individuals and communities in contexts characterized by institutional gaps (Kolk, 2014). Also, as the use of political economic strategies that advocate using more effectively the capacities of small landowners for rural development and well-being through the promotion of small-scale production systems (Pokorny et al., 2013).

In relation to social capital, we find the following: the contribution of micro-entrepreneurship has played a prominent role in the development of employment around the world; however, entrepreneurs must consider the financial, human, and social capital before starting their activity, as well as the hostility of the environment (Vial, 2011). For example, microfinance could help improve the well-being of clients and maximize business results (Newman et al., 2014).

Some research calls, therefore, for a more collective vision of management that is based on trust and social well-being, as greater well-being generates more cohesive, productive, and happy societies (Zhao and Roper, 2011). Likewise, other factors influence happiness, such as asset ownership, family ties, personal attributes, characteristics of households, human, and social capital, financial security, labor relations, and participation of the community (Mahadea and Ramroop, 2015).

**Figure 3:**
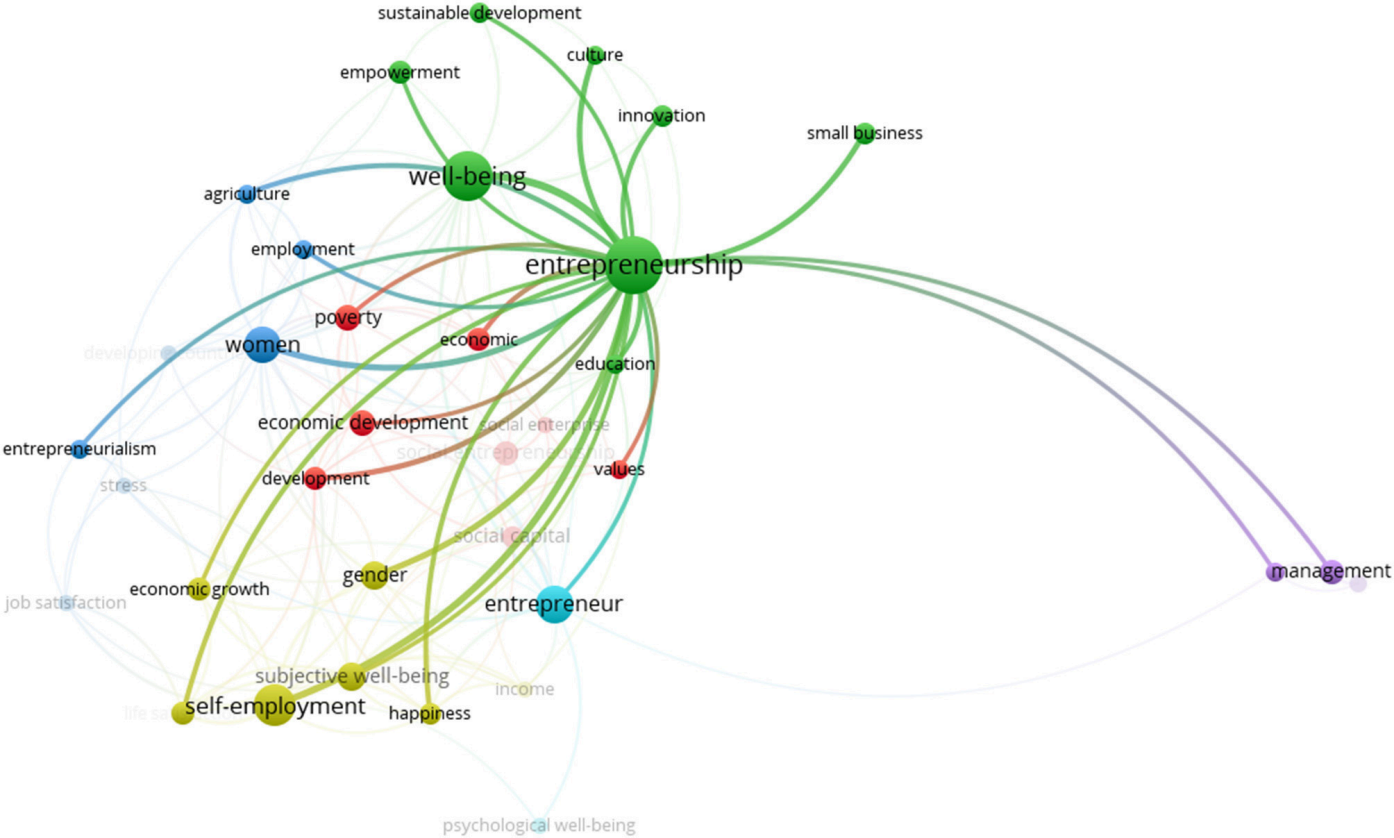
Relations of the green cluster.

The green cluster is shows the closeness and link strength in the words “entrepreneurship” and “well-being.” Associated with these two keywords are culture, education, empowerment, innovation, small business and sustainable development. The 27.30% occurrence of keywords under study are related to this cluster it is the one that concentrates the greatest amount of relations with the rest of the clusters, as shown in Figure 3.

Additionally, the advancement of research into entrepreneurs' well-being from the perspective of culture and innovation starts very early, given the interest in improving people's well-being within society (Dawson and Daniel, 2010) and the community culture that promotes development businesses (Huggins and Thompson, 2014).

For example, subsistence entrepreneurs in developing countries carry out vital marketing activities, overcome substantial life challenges, and improve their economic capacity and that of their communities (Sridharan et al., 2014). This keywords, are associated as well with research that considers the importance of inventor-entrepreneurs, whose potential lies in their possibility of growing and societal well-being (Miner et al., 1992).

Regarding these, we found the following: first, at the absence of financial rewards, motivation for achievement does not improve the will to grow unless it is intrinsically motivated (Davidsson, 1989). Second, coping based on cognitive response facilitates well-being and business performance (Drnovsek et al., 2010; Uy et al., 2013).

Third, employers, compared to employees, show higher levels of happiness (Mahadea and Ramroop, 2015). Fourth, to achieve greater well-being and productivity, it is necessary to carry out social and leisure activities based on hobbies, since relaxation and rest are not enough to achieve real well-being (Shen et al., 2018).

The third cluster in blue associates the following keywords: agriculture, developing countries, employment, entrepreneurialism, job satisfaction, stress and women. The 13.15% occurrence of keywords under study are related to this cluster.

The variable “women” was found to have been associated with the construct entrepreneurship since the beginning of research into entrepreneurs' well-being, because it is thought that women can be managers of a positive change in their personal, social, and economic well-being, and a positive influence on their immediate community (Sridharan et al., 2014).

Also associated with this cluster are certain financial tools, such as micro-credits, that can be effective in alleviating poverty (Ang, 2004) in order to obtain better access to markets to improve entrepreneurs' well-being in a sustainable manner, despite limitations (Kolk, 2014).

Interestingly, the report of the GEN (2017–2018) reveals that the proportion of participation of men and women in entrepreneurial activity, in the initial stage, varies considerably and is reflected in cultural differences and customs with respect to women. Generally, in economies driven by factors, such as efficiency and innovation, women will have an entrepreneurial participation motivated by necessity, and inferior to that of men.

The fourth cluster in yellow associates the following keywords: economic growth, gender, happiness, income, life satisfaction, self-employment, and subjective well-being. The 10.33% occurrence of keywords under study are related to this cluster.

From research that has focused on the search for entrepreneurial well-being in the aspect of “self-employment” and its relationship with gender, we have considered the following findings:

Individuals who are self-employed enjoy greater autonomy, have flexibility at work and report higher levels of participation in work and job satisfaction than those employed by organizations (Parasuraman and Simmers, 2001). Despite lower incomes, self-employed workers consistently report higher job satisfaction (Binder and Coad, 2013).

However, it is also true that self-employed workers experience higher levels of work–family conflict and lower family satisfaction compared to employees of organizations (Parasuraman and Simmers, 2001). Work–family conflict has a direct negative effect on mental health, work, family, and life satisfaction (Nguyen and Sawang, 2016); additionally, high job satisfaction can make entrepreneurs neglect other important domains of life, as gratifying work excludes other pleasures (Binder and Coad, 2013). When the entrepreneur's experience in the operation helps them improve their quality of life and family well-being, they are less likely to consider leaving the business (Hsu et al., 2016).

Self-employment is also positively related to subjective well-being, despite the differences between groups of self-employed workers. For example, self-employed workers report a higher level of life satisfaction compared to self-employed workers without employees. Immigrants experience greater life satisfaction compared to natives (Johansson Sevä et al., 2016).

We also find that being unemployed produces a much stronger decline in life satisfaction for self-employed workers than for paid employees (Hetschko, 2016). People who exchange regular employment for self-employment experience an increase in life satisfaction (up to 2 years later), while those who move from unemployment to self-employment are no more satisfied than their counterparts who go from unemployment to regular employment (Binder and Coad, 2013).

Finally, it is said that entrepreneurs, to solve their problems, can oscillate between taking active measures (active coping), or temporarily distance (i.e., avoiding confrontation).The effective use of avoidance coping improves immediate psychological well-being, since incorporating short breaks and temporary respite can be beneficial. In the long term, employers must use their ability to avoid issues, along with active coping, and learn to take advantage of both methods to deal with problems (Uy et al., 2013).

The fifth cluster in turquoise associates the following keywords: entrepreneur and psychological well-being. The 22.35% occurrence of keywords under study are related to this cluster. Although the number of words is low, it is the second cluster with the most relationships with the others.

This cluster relates those studies where psychological aspects acquire an important role in entrepreneurship. For example, people who are willing to take short-term risks are more likely to start a business, as are those who have high psychological well-being (Zhang et al., 2015) and if they also have control of the environment, self-acceptance and autonomy, a good financial performance is foreseen (Farrington, 2017). In addition, openness to experience, autonomy and persistence in terms of goal pursuit increases the likelihood that a person will seek self-employment, while neuroticism (Patel and Thatcher, 2014) and being a low-skilled worker (Lofstrom, 2013) reduce the likelihood of entrepreneurship.

These variables have also been used when making comparisons between entrepreneurs and non-entrepreneurs, indicating that it is possible that sometimes employers show lower returns than non-entrepreneurs do. However, this is compensated by non-pecuniary benefits, such as greater control over their work environment, greater optimism, and social capacity (Shefrin, 2011). In conclusion, entrepreneurs show greater well-being and better physical, mental and behavioral conditions (Stephan and Roesler, 2010).

Finally, the last of the clusters in purple, is located on the right side and shown in Figure 2, is far from the main group. This reflects the low strength of the relationship with the rest of the elements (van Eck and Waltman, 2010). The following keywords are associated with this cluster: business, management and psychology, applied. The 9.2% of the keywords relate to this cluster.

It deals with aspects related to business and its administration, thus, although well-being of entrepreneurs is present in this topic, it interacts weakly in our cluster relationship model. For example, there is mention of the metropolization process that has privileged a handful of dynamic urban centers but, on the other hand, the need for governments to activate rural areas, thus improving the well-being of the population in general (Smetkowski, 2013).

Also aspects related to different types of administration, such as that of the founders of family firms that experienced higher levels of support from other family businesses than those who owned non-family businesses, with additional support provided to the former in terms of social well-being and resilience via the family's integration into business (Powell and Eddleston, 2017).

##### Bibliometric review

The theme of “well-being” has been extensively discussed. For example, Mandell (1974) states in his article “The Changing Facet of the Chair of Psychiatry Departments in America: An Opinion,” as “two decades ago psychiatrists were trained by a few charismatic, humanistic clinicians who did not have much to do with laboratories or fund raising. After World War II academic psychiatry was transformed, largely by federal money, into a multidimensional scientific enterprise the leaders of which needed to be scientific entrepreneurs as well as persuasive humanists” (Mandell, 1974, p. 1137) so that universal well-being was relegated to a second plane.

The next group of articles found relate to social problems, such as public transport service as a vital operation for the well-being of cities (Grava, 1980), policy statements between governments in search of the cities' well-being, and small businesses as a means of generating employment (Rothwell, 1984). We also found a study on the inequality of the black population. It concludes that “despite renewed governmental encouragement and some real growth, black-owned businesses still represent a trivial fraction of total businesses; and the black share of business firms in a city seems to have a substantial impact on the relative well-being of blacks in that city” (Villemez and Beggs, 1984, p. 137).

**Figure 4:**
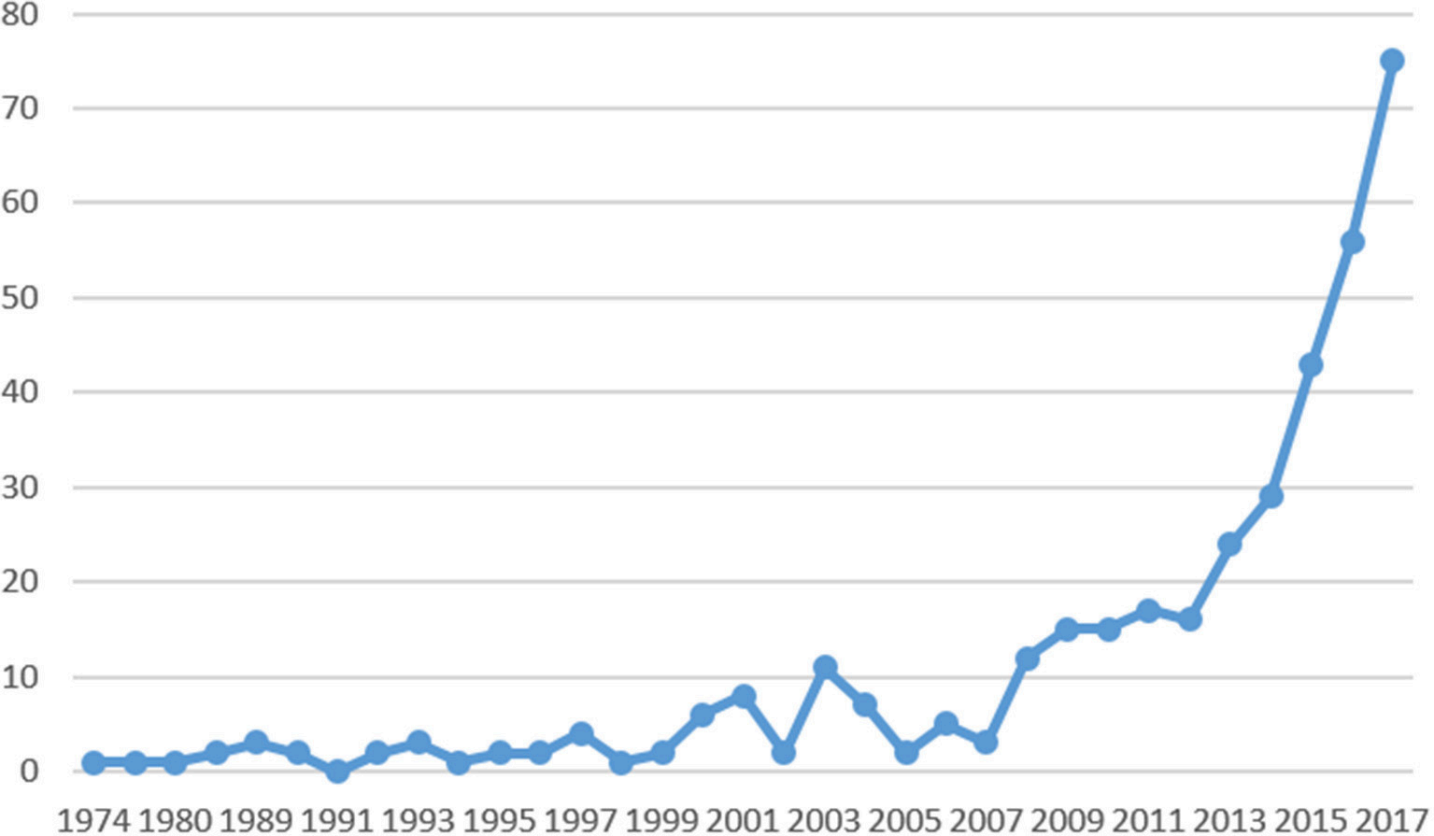
Frequency of publications by year.

On recent studies, entrepreneur and well-being acquires a different connotation. As seen in Figure 4, from 2009 onwards the number of articles published in high-impact journals increases exponentially, revealing the importance that researchers have conferred on the subject.

**Figure 5:**
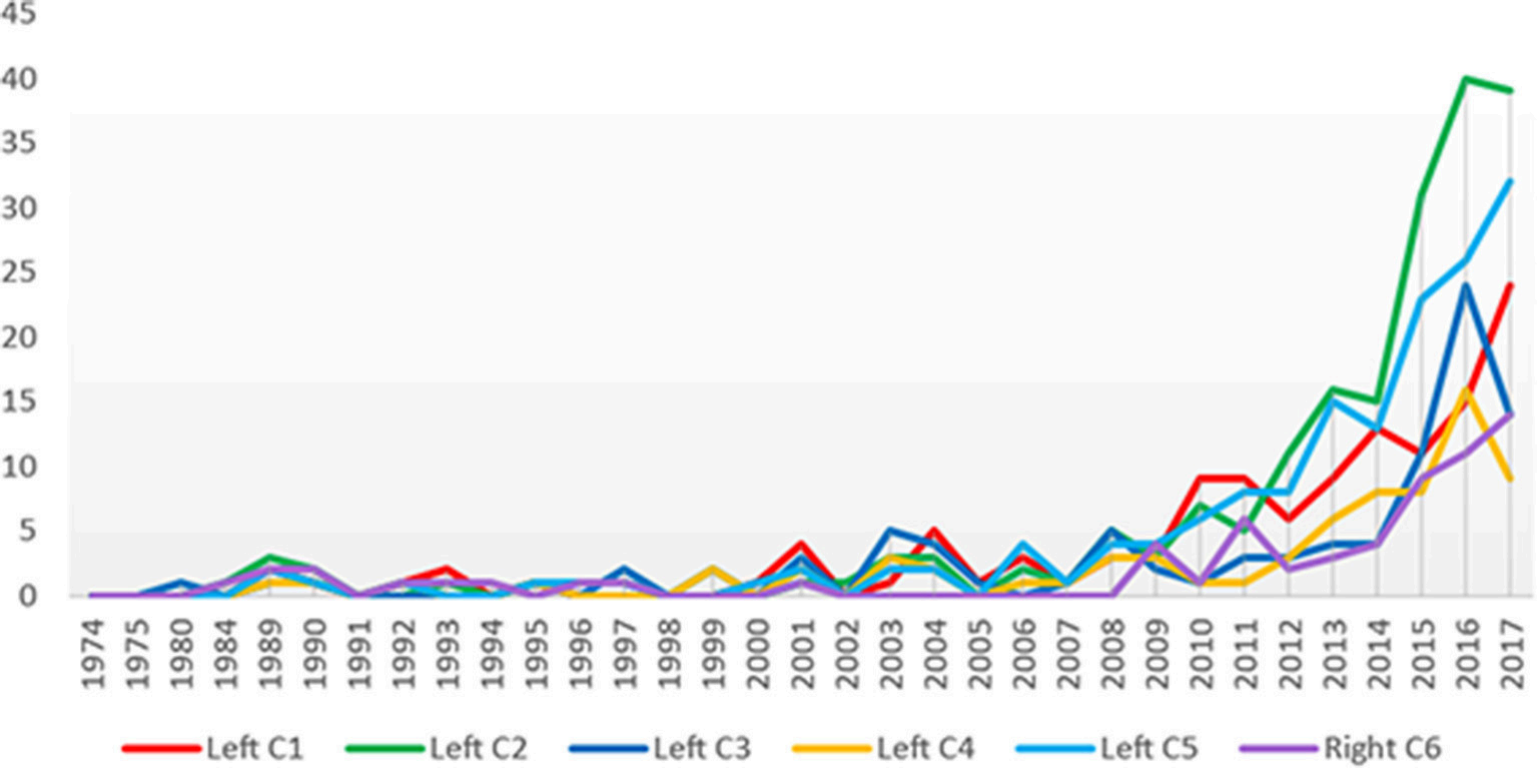
Article published by cluster by year.

As observed, the “well-being” construct is associated with many fields of knowledge, as is “entrepreneur ^*^.” In Figure 5, we have made a comparison of the progress in the research from 1984 to 2017, associated with the extracted clusters, in order to visualize the knowledge growth in those areas that has been of greater focus during this period.

For example, in recent years, the green line that relates entrepreneur well-being with culture, education and innovation is the one that shows the most growth, followed by the turquoise line, which, as we studied, is associated with the psychological aspects that allow the entrepreneur to achieve well-being.

Subsequently, the red and blue lines are presented. The first is related to social entrepreneurship and maintains a constant growth, and the second with the problem of women, which shows a decrease in the last year.

Finally, we find the lines in yellow and purple. The first, related to self-employment, shows a decrease in the last year and the purple, whose link with entrepreneurial well-being is less strength, shows a more conservative growth.

##### Relationships between journals

**Figure 6:**
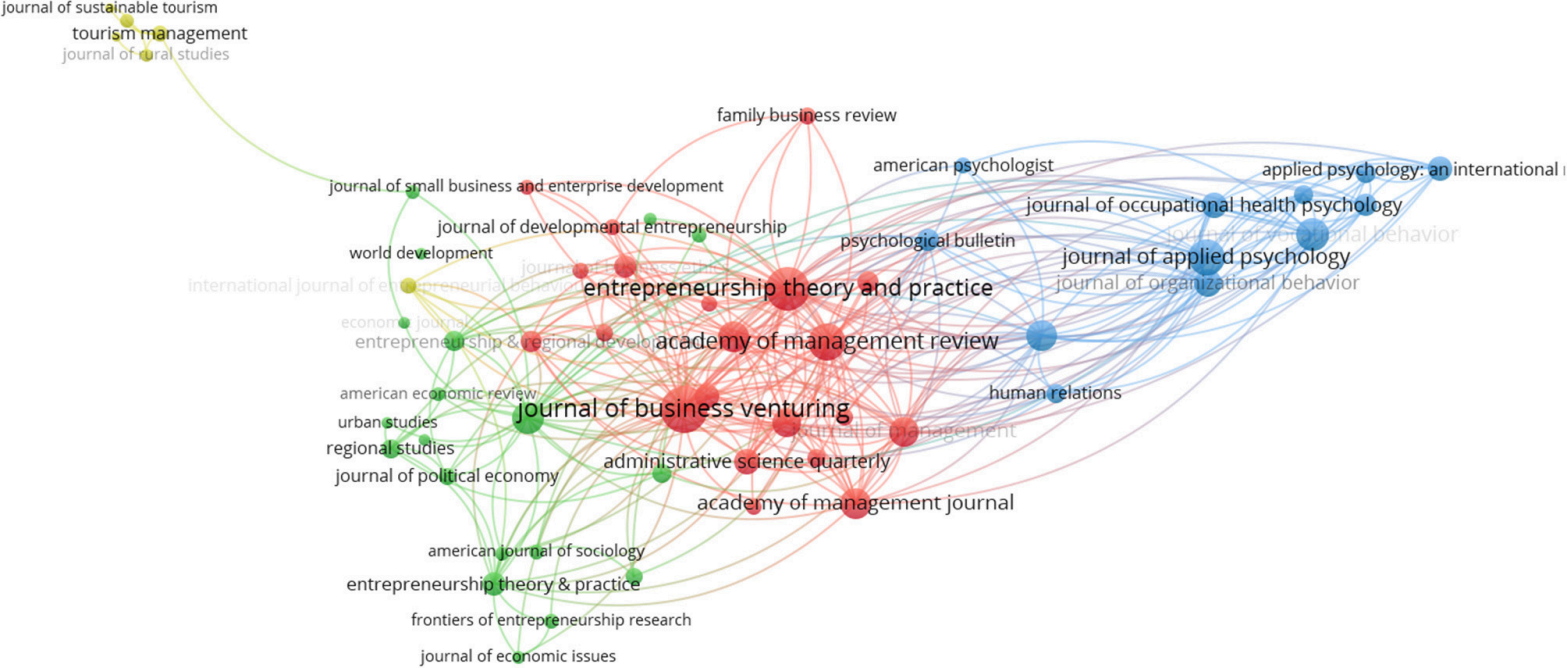
Clusters of the journals.

To identify which journals have the most cited articles associated with the issue of the entrepreneurs' well-being, we analyzed the data with a pre-specified minimum number of citations per source of 20. Then the VOSviewer program selected 58 sources, whose relationships are shown in Figure 6.

Clearly, four clusters are distinguished: from left to right, the first and smallest related to journals that publish on sustainable or tourism studies. Followed by the green, that frames journals related to the economic field; network, whose relationships are stronger and mediate the cluster, related to issues of business administration and the fourth cluster in blue, related to psychology issues.

The first 10 journals with the highest citation number and with the strongest link, in descending order, are: Journal of Business Venturing, Entrepreneurship Theory and Practice, Academy of Management Review, Journal of Applied Psychology, Journal of Vocational Behavior, Small Business Economics, Journal of Personality and Social Psychology, Academy of Management Journal, Journal of Small Business Management and Journal of Management. All of them classified between the first and second quartiles in 2016.

##### Relationship between authors

**Figure 7:**
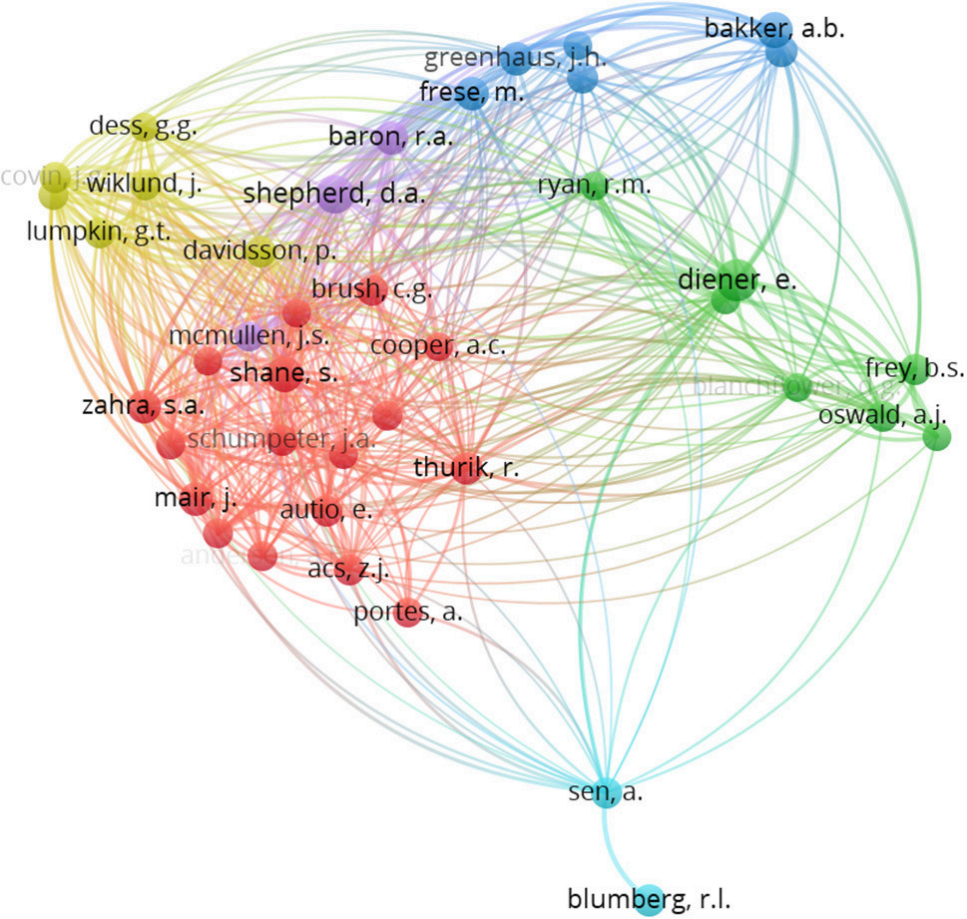
Clusters of the citation of authors.

To identify which authors that have the most cited articles associated with the topic of entrepreneur' well-being, we analyzed the data with a pre-specified minimum number of citations per source of 20. Then VOSviewer program selected 29 authors, whose relationships are shown in Figure 7.

Mapping and clustering denotes the number of links, in this case, co-citation links. This can be interpreted in terms of attractive and repulsive forces between nodes. “The higher the association strength of two nodes, the stronger the attractive force between the nodes. Since the strength of the repulsive force between two nodes does not depend on the association strength of the nodes, the overall effect of the two forces is that nodes with a high association strength are pulled toward each other while nodes with a low association strength are pushed away from each other” (van Eck and Waltman, 2010, p. 631).

On the other hand, in the “full counting” option, the highly cited references are considered more representative, but not in the “fractional counting” option in which each reference cited in a publication has the same influence, or is considered equally representative in the publication. So, to carry out this analysis, the “fractioned counting” was considered, so each reference cited in a publication has the same representativeness, determining in the entrepreneurial well-being field, which are the authors with the most influence.

Figure 7 shows 6 clusters, whose largest group comprises of 17 authors, the following 5 clusters on average has a size of 4.80 with a standard deviation of 1.94. A summary of the contents of the five groups is provided in Tables 2–7.

**Table 2:** Cluster 1.

	**Cluster 1 author**	**#Cita**	**T. link strength**	**Example title**	**Cluster keywords**	**Keywords associated with author's citation**
1	Shane S	44	41,87	“The Promise of entrepreneurship as a field of research” (Shane and Venkataraman, 2000)	Left (1,2,3,4,5) Rigth (6)	Development; economic; social capital; social enterprise; social entrepreneurship; values; culture; education; innovation; employment; entrepreneurialism; women; economic growth; gender; happiness; life satisfaction; self-employment; psychological well-being; business; management
2	Zahra SA	33	29,65	“On the Frontiers: The Implications of Social Entrepreneurship for International Entrepreneurship” (Zahra et al., 2014)	Left (1,2,3,4,5) Rigth (6)	Development; economic; social enterprise; social entrepreneurship; values; culture; innovation; small business; developing countries; entrepreneurialism; women; economic growth; business; management
3	Mair J	36	28,26	“Social entrepreneurship: Creating new business models to serve the poor” (Seelos and Mair, 2005)	Left (1,2,3,4,5) Rigth (6)	Development; economic; poverty; social capital; social enterprise; social entrepreneurship; values; innovation; sustainable development; employment; women; gender; psychological well-being; business; management
4	Thurik R	30	26,66	“Linking entrepreneurship and economic growth” (Thurik and Wennekers, 1999)	Left (1,2,3,4,5) Rigth (6)	Development; economic; social capital; social enterprise; social entrepreneurship; values; innovation; employment; job satisfaction; women; gender; happiness; life satisfaction; self-employment; psychological well-being; business; management
5	Schumpeter JA	27	24,11	“The Theory of Economic Development” (Schumpeter, 1934)	Left (1,2,3,4,5) Rigth (6)	Development; economic; social capital; social enterprise; social entrepreneurship; culture; education; innovation; small business; developing countries; employment; entrepreneurialism; women; economic growth; gender; happiness; self-employment; psychological well-being; business; management; psychology, applied
6	Autio E	28	23,17	“Social capital, knowledge acquisition, and knowledge exploitation in young technology-based firms” (Yli-Renko et al., 2001)	Left (1,2,3,4,5) Rigth (6)	Development; economic; social entrepreneurship; education; innovation; employment; women; economic growth; happiness; life satisfaction; self-employment; business; management
7	Acs ZJ	25	19,71	“Entrepreneurship, agglomeration and technological change” (Acs and Varga, 2005)	Left (1,2,3,4,5)	Development; economic; poverty; social entrepreneurship; culture; innovation; employment; entrepreneurialism; job satisfaction; women; economic growth; gender; happiness; life satisfaction; self-employment
8	Reynolds PD	22	19,5	“Who starts new firms?–Preliminary explorations of firms-in-gestation” (Reynolds, 2013)	Left (1,2,3,4,5) Rigth (6)	Development; economic; social capital; social entrepreneurship; values; culture; education; innovation; agriculture; developing countries; employment; entrepreneurialism; women; economic growth; happiness; life satisfaction; self-employment; psychological well-being; business; management
9	Carter, S	20	19,42	“The Rewards of Entrepreneurship: Exploring the Incomes, Wealth, and Economic Well-Being of Entrepreneurial Households” (Carter, 2011)	Left (1,2,3,4,5) Rigth (6)	Development; economic; social entrepreneurship; values; culture; education; empowerment; innovation; small business; agriculture; employment; entrepreneurialism; job satisfaction; stress; women; economic growth; gender; self-employment; psychological well-being; business; management
10	Westhead, P	20	19,21	“Opportunity Identification and Pursuit: Does an Entrepreneur's Human Capital Matter?” (Ucbasaran et al., 2008)	Left (1,2,3,4,5) Rigth (6)	Development; economic; social entrepreneurship; values; culture; innovation; small business; entrepreneurialism; economic growth; psychological well-being; business; management; psychology, applied
11	Venkataraman	20	18,97	“Aspirations, Market Offerings, and the Pursuit of Entrepreneurial Opportunities” (Lee and Venkataraman, 2006)	Left (1,2,3,4,5) Rigth (6)	Development; economic; social capital; social enterprise; social entrepreneurship; values; education; innovation; entrepreneurialism; economic growth; gender; happiness; life satisfaction; psychological well-being; business; management
12	Welter, F.	20	18,75	“Extending Women's Entrepreneurship Research in New Directions” (Hughes et al., 2012)	Left (1,2,3,4,5) Rigth (6)	Development; economic; social capital; innovation; employment; entrepreneurialism; job satisfaction; women; economic growth; gender; life satisfaction; self-employment; business
13	Cooper, A.C	20	18,26	“Determinants of satisfaction for entrepreneurs”(Cooper and Artz, 1995)	Left (1,2,3,4,5) Rigth (6)	Development; economic; poverty; social entrepreneurship; values; culture; education; empowerment; innovation; small business; sustainable development; agriculture; employment; entrepreneurialism; job satisfaction; stress; women; economic growth; gender; happiness; income; life satisfaction; self-employment; psychological well-being; business; management; psychology, applied
14	Hofstede G	22	16,25	“Measuring organizational cultures: A qualitative and quantitative study across twenty cases” (Hofstede et al., 1990)	Left (1,2,3,4,5) Rigth (6)	Development; economic; social entrepreneurship; values; culture; education; empowerment; innovation; small business; agriculture; employment; entrepreneurialism; job satisfaction; stress; women; economic growth; gender; self-employment; psychological well-being; business; management
15	Anderson. A.R	24	16,15	“Ambivalence and Ambiguity in Social Enterprise; Narratives about Values in Reconciling Purpose and Practices” (Diochon and Anderson, 2011)	Left (1,2,3,4,5) Rigth (6)	Development; economic; poverty; social capital; social enterprise; social entrepreneurship; values; culture; education; empowerment; innovation; small business; sustainable development; agriculture; employment; entrepreneurialism; stress; women; economic growth; gender; income; self-employment; business; management
16	Brush CG	25	15,84	“Advancing a framework for coherent research on women's entrepreneurship” (De Bruin et al., 2007)	Left (1,2,3,4,5) Rigth (6)	Development; economic; poverty; social entrepreneurship; values; empowerment; innovation; small business; employment; entrepreneurialism; job satisfaction; stress; women; gender; self-employment; business; psychology, applied
17	Portes, A	24	6,6	“Gaining the Upper Hand: Economic Mobility among Immigrant and Domestic Minorities” (Portes and Zhou, 1992)	Left (1,2,3,4,5) Rigth (6)	Development; economic; poverty; social capital; social entrepreneurship; culture; education; small business; employment; self-employment; psychological well-being; business

**Table 3:** Cluster 2.

	**Cluster 2 author**	**#Cita**	**T. link strength**	**Example title**	**Cluster keywords**	**Keywords associated with author's citation**
1	Diener E	76	57,16	“Beyond the hedonic treadmill: Revising the adaptation theory of well-being” (Diener et al., 2006)	Left (1,2,3,4,5) Rigth (6)	Development; economic; social capital; values; culture; education; small business; employment; entrepreneurialism; job satisfaction; stress; women; gender; happiness; income; life satisfaction; self-employment; business; management; psychology, applied
2	Frey, B.S	25	21,9	“Being Independent Is a Great Thing: Subjective Evaluations of Self-Employment and Hierarchy” (Benz and Frey, 2008)	Left (1,2,3,4,5) Rigth (6)	Development; economic; social capital; culture; education; employment; entrepreneurialism; job satisfaction; women; economic growth; gender; happiness; income; life satisfaction; self-employment; business; management
3	Oswald A.J	25	21,53	“Satisfaction and comparison income” (Clark and Oswald, 1996)	Left (1,2,3,4,5) Rigth (6)	Development; economic; social capital; culture; education; employment; entrepreneurialism; job satisfaction; gender; happiness; income; life satisfaction; self-employment; business; management
4	Lucas, R.E	20	17,24	“Four Myths about Subjective Well-being” (Lucas et al., 2008)	Left (1,2,3,4,5) Rigth (6)	Development; economic; values; education; small business; employment; job satisfaction; women; gender; happiness; income; self-employment; psychological well-being; business
5	Blanchflower D.G	20	16,82	“Unemployment, well-being, and wage curves in eastern and central Europe” (D G Blanchflower, 2001)	Left (1,2,3,4,5) Rigth (6)	Development; economic; culture; education; employment; entrepreneurialism; job satisfaction; gender; happiness; income; life satisfaction; self-employment; psychological well-being; business
6	Stutzer, A.	20	16,47	“Latent entrepreneurship across nations” (Blanchflower et al., 2001)	Left (1,2,3,4,5)	Development; economic; social capital; values; education; employment; job satisfaction; gender; happiness; income; life satisfaction; self-employment
7	Ryan RM	20	15,36	“On Happiness and Human Potentials: A Review of Research on Hedonic and Eudaimonic Well-Being” (Ryan and Deci, 2001)	Left (1,2,3,4,5) Rigth (6)	Development; economic; values; empowerment; employment; stress; women; economic growth; gender; income; self-employment; psychological well-being; business; management

**Table 4:** Cluster 3.

	**Cluster 3 author**	**#Cita**	**T. link strength**	**Example title**	**Cluster keywords**	**Keywords associated with author's citation**
1	Frese M	33	28,01	“Happy and Proactive? The Role of Hedonic and Eudaimonic Well-Being in Business Owners' Personal Initiative” (Hahn et al., 2012)	Left (1,2,3,4,5) Rigth (6)	Development; economic; culture; innovation; small business; agriculture; employment; job satisfaction; stress; women; economic growth; gender; life satisfaction; self-employment; psychological well-being; business; psychology, applied
2	Bakker AB	43	26,19	“Work engagement: An emerging concept in occupational health psychology” (Bakker et al., 2008)	Left (1,2,3,4,5) Rigth (6)	Development; economic; culture; agriculture; employment; entrepreneurialism; stress; women; gender; happiness; income; life satisfaction; self-employment
3	Schaufeli WB	32	24,01	“The measurement of engagement and burnout: A two sample confirmatory factor analytic approach” (Schaufeli et al., 2002)	Left (1,2,3,4,5) Rigth (6)	Development; culture; agriculture; entrepreneurialism; stress; women; gender; psychological well-being; business
4	Greenhaus JH	33	22,09	“When work and family are allies: A theory of work-family enrichment” (Greenhaus and Powell, 2006)	Left (1,2,3,4,5) Rigth (6)	Development; culture; small business; entrepreneurialism; job satisfaction; stress; women; gender; psychological well-being; business; management; psychology, applied
5	Parasuraman S	26	20,47	“Work and Family Variables, Entrepreneurial Career Success, and Psychological Well-Being” (Parasuraman et al., 1996)	Left (1,2,3,4,5) Rigth (6)	Development; economic; culture; small business; employment; job satisfaction; stress; women; economic growth; gender; life satisfaction; self-employment; psychological well-being; business; management; psychology, applied
6	Stephan, U	21	18,39	Advancing the Psychology of Entrepreneurship: A Review of the Psychological Literature and an Introduction (Gorgievski and Stephan, 2016)	Left (1,2,3,4,5) Rigth (6)	Economic; small business; employment; job satisfaction; women; economic growth; gender; life satisfaction; self-employment; psychological well-being; business; psychology, applied

**Table 5:** Cluster 4.

	**Cluster 4 author**	**#Cita**	**T. link strength**	**Example title**	**Cluster keywords**	**Keywords associated with author's citation**
1	Wiklund J	28	25,48	“The Age-Effect of Financial Indicators as Buffers against the Liability of Newness” (Wiklund et al., 2010)	Left (1,2,3,4,5) Rigth (6)	Economic; social enterprise; values; employment; entrepreneurialism; women; economic growth; self-employment; psychological well-being; business; management
2	Lumpkin GT	26	24,54	“Clarifying the entrepreneurial orientation construct and linking it to performance” (Lumpkin and Dess, 1996)	Left (1,2,3,4,5) Rigth (6)	Social enterprise; social entrepreneurship; values; innovation; employment; job satisfaction; women; gender; life satisfaction; self-employment; psychological well-being; business; management
3	Covin JG	27	23,89	“Strategic management of small firms in hostile and benign environments” (Covin and Slevin, 1989)	Left (1,2,3,4,5) Rigth (6)	Development; economic; social enterprise; values; innovation; entrepreneurialism; economic growth; psychological well-being; business; psychology, applied
4	Davidsson P	25	23,22	“Entrepreneurship - And after? A Study of Growth Willingness in Small Firms” (Davidsson, 1989)	Left (1,2,3,4,5) Rigth (6)	Development; economic; social capital; social enterprise; values; small business; employment; entrepreneurialism; women; economic growth; gender; life satisfaction; self-employment; psychological well-being; business; management; psychology, applied
5	Slevin DP	22	19,93	“Entrepreneurship and the concept of fit: A model and empirical tests” (Naman and Slevin, 1993)	Left (1,2,5) Rigth (6)	Social enterprise; values; innovation; management
6	Dess GG	20	18,8	“Clarifying the entrepreneurial orientation construct and linking it to performance” (Lumpkin and Dess, 1996)	Left (1,2,3,4,5) Rigth (6)	Values; women; gender; management; psychology, applied

**Table 6:** Cluster 5.

	**Cluster 5 author**	**#Cita**	**T. link strength**	**Example Title**	**Cluster Keywords**	**Keywords associated with author's citation**
1	Shepherd DA	54	46,21	“Birds of a Feather Don't Always Flock Together: Identity Management in Entrepreneurship” (Shepherd and Haynie, 2009)	Left (1,2,3,4,5) Rigth (6)	Development; economic; social enterprise; values; education; sustainable development; employment; entrepreneurialism; women; economic growth; gender; happiness; life satisfaction; self-employment; psychological well-being; business; management
2	Baron RA	35	30,28	“Why entrepreneurs often experience low, not high, levels of stress: The joint effects of selection and psychological capital” (Baron et al., 2016)	Left (1,2,3,4,5) Rigth (6)	Social enterprise; social entrepreneurship; values; education; innovation; small business; employment; stress; women; gender; self-employment; psychological well-being; business; management
3	Mcmullen J.S.	24	21,32	“Social Entrepreneurship and the Development Paradox of Prosocial Motivation: A Cautionary Tale” (McMullen and Bergman, 2017)	Left (1,2,3,4,5) Rigth (6)	Development; economic; social capital; social entrepreneurship; innovation; sustainable development; employment; entrepreneurialism; economic growth; self-employment; business

**Table 7:** Cluster 6.

	**Cluster 6 author**	**#Cita**	**T. link strength**	**Example Title**	**Cluster Keywords**	**Keywords associated with author's citation**
1	Sen A	28	15,56	“Development as Freedom” (Sen, 2000)	Left (1,2,3,4,5) Rigth (6)	development; economic; poverty; social capital; social entrepreneurship; values; culture; education; empowerment; sustainable development; employment; women; economic growth; gender; happiness; income; life satisfaction; self-employment; psychology, applied;
2	Blumberg RL	31	3,26	“We are family”: Gender, microenterprise, family work, and well-being in Ecuador and the Dominican Republic—with comparative data from Guatemala, Swaziland, and Guinea-Bissau (Blumberg, 2001)	Left (1,2,3,4,5)	development; economic; poverty; culture; education; empowerment; innovation; agriculture; women; gender;

It is important to mention that all authors considered in the following tables have been cited in issues related to entrepreneur's well-being; therefore, both terms and derivations have not been included in the tables.

## Results

### Synthesized findings

The articles were identified using the terms “well-being” and “entrepreneur^*^” and their derivations. We eliminated all studies that were irrelevant or repeated; whose keywords were not within the abstract, keywords, or title; or were not the correct document type (e.g., book chapters) (see Table 1). The study from the 373 articles was carried out using two analysis strategies: first the clusters are analyzed, the conformation of the clusters is presented in all areas where well-being is considered within entrepreneurship activities, and its topics reported. In the second, a bibliometric study was carried out, in which indicators are described: where is it published, years in which has been published, growth, and author's citation.

From the general analysis, topics identified in relation to the terms “well-being” and “entrepreneur^*^” were found to be very diverse, and related to aspects of health, psychology, economy, society, and culture, at both the micro (individual) and macro (associated with communities or countries) level.

In the cluster analysis, the six cluster and the relationships between them are described. Mainly related with: culture, education, innovation, sustainable development and small business; psychological well-being; social entrepreneurship and economic development; women and employment; and self-employment; life satisfaction and economic growth, and business administration.

Additionally, as shown in Figure 4, research on entrepreneurs' well-being has grown considerably, especially in the last decade, and the issue has been an increasing focus of governments, communities, and societies in general.

Finally, it is important to note that entrepreneurs' well-being has been discussed in rigorous and high-quality scientific journals, guaranteeing article and finding's reliability, also that there is a high number of authors, who are frequently cited, and whose academic experience in the area of entrepreneurship is well-recognized. An index of collaboration per article of 1.89 was obtained (704 authors participated in the 373 articles studied).

### Risk of bias

Our research collected information on entrepreneurs' well-being from SSCI, Scopus and ProQuest. These three databases include articles based on quality and academic relevance. Therefore, an assessment of the method used in each study to determine the integrity of the research is not required, since any risk was eliminated by using these particular databases. On the other hand, once we began to extract the information, we followed a specific protocol and established objectives, setting aside any personal criteria that may have hindered the investigation. Thus, in the first part, article relationships were analyzed according to bibliographic maps based on distance, and in the second part, other aspects were analyzed such as: citation, sources, and years of publication.

Finally, any disagreement between the authors of this study that may have led to bias was resolved through discussion and with the participation of the third author when necessary.

## Discussion and conclusions

### Summary of main findings

According to GEM (2017–2018) the levels of entrepreneurship have a growing trend and also, a considerable percentage, has planned to start some activity in the near future. However, we have argued that well-being functions as an engine in the life of the entrepreneur, because in order for companies to maintain themselves over time it is necessary for entrepreneurs to recognize the benefits of the activity. These benefits can be classified according to the aspirations and motivations of the entrepreneur, however it all comes down to the idea implicit to the human being of wanting to obtain maximum well-being. Therefore, this bibliometric review summarizes and structures in a general way (Perianes-Rodriguez et al., 2016), a large number of articles, in order to better understand the link between the entrepreneur and his well-being.

We made sure that the quality of the information was optimal and we could answer the research questions: In which areas of study does the well-being of the entrepreneur acquire relevance? What has been its importance over time? Who are the authors? mostly cited? and What are the sources that are mostly consulted?

On the basis of a formal review of 373 articles selected and published in research journals, an analysis of the literature that shows the underlying structure of its different research streams was carried out. Our study identified:

- That there were many areas of study where the well-being of entrepreneurs acquires relevance, however, according to their relationship and closeness, they were grouped into six theoretical perspectives. The one that obtained the highest percentage of relationships is associated with culture, education, innovation, sustainable development, and small businesses, followed by the cluster of psychological well-being.
- Later in a similar category, there are the clusters that are related to social entrepreneurship and economic development; women and employment, and self-employment, satisfaction with life and economic growth. Finally, we find the last cluster, which maintains a distant relationship and corresponds to business administration.
- That the topic has been analyzed by researchers of academic trajectory, who have expressed their contribution to the field.
- That the topic has been published in journals with a high impact factor.
- That in the last decade there has been considerable growth in publications related to entrepreneurs' well-being.

These previous points allow to fundament the importance of the entrepreneurial well-being topic in entrepreneurial initiatives, and the increasing amount of research justifies this need for a bibliometric analysis, providing a knowledge structure that had been, until now, confusing.

Our findings show that the entrepreneur's well-being is an important construct that has been used in a common body of theoretical statements and applied to different contexts. Several approaches characterize this literature, such as economic sciences, psychology, and sociology playing an important role. Hundreds of published articles recognize the role of the entrepreneur and value their subjective well-being.

From a practical perspective, our research contributes the theory construction and teaching. First, academics can now position their work in the field, as well as identify possible new issues and gaps that can be used to formulate new research questions. Some possible contributions to the existing literature have been detected and could be considered useful in the new effort to integrate the well-being into entrepreneurship. For example, to study whether re-education in entrepreneurs could improve their attitude, and with it, their well-being. A transformation in the way of thinking could modify entrepreneurial behavior.

In addition, this research could be useful for those who work in entrepreneurship and who have no experience in the theoretical fields reviewed above (culture and innovation, self-employment, women, capital and social entrepreneurship, economic development and management), as well as the comprehension in the amplitude of the constructs “well being and entrepreneurial.” As indicated above, the definition of “well being,” as well as that of “entrepreneur,” is adapted to the objectives and criteria of the researcher. In this way, different definitions of the terms were found in the literature and, therefore, the measurements and conclusions were adjusted to the criteria used.

Second, entrepreneurs can have an overview of the area in which they operate and benefit from some of the conclusions reached by researchers, for example, how to face firm problems (Uy et al., 2013). In addition, on rethinking strategies in order to achieve a greater advantage in the market or improve positioning by analyzing well-being contributors, whether hedonic (Diener et al., 1999), eudaimonic (Ryan and Frederick, 1997; Ryan and Deci, 2001), or inherent to an entrepreneurial activity (Rotemberg-Shir, 2015).

Aspirations could affect an entrepreneur's well-being. Studies have shown that extrinsic objectives are negatively related to well-being (Ilardi et al., 1993; Sheldon et al., 2004), however, economic aspects move many entrepreneurs, obtaining wealth or fame, which, aspirations, and motivations that may have an entrepreneur could lead to the detriment of their well-being.

Third, teachers can use this research to present a general structure and development on entrepreneur's well-being, by identifying some of the most relevant aspects mentioned. Fourth, government ministers may find this research beneficial when they visualize well-being in entrepreneurship as an opportunity to improve the country's economy or community development (Samli, 2008; Harriss-White, 2010).

Finally, as expected by Shane and Venkataraman (2000), it is now possible to consolidate a set of systematic information on entrepreneurship that, not only allows to observe the field's progress, but also provides information about entrepreneur's well-being, in order that growth is assured through the different entrepreneur's stages.

Given that well-being is as much desired by entrepreneurs as it is by people in general, and considering that there are many studies whose ultimate goal is to achieve this status, possibly the greatest contribution of this research at a practical level is to motivate continuing education toward good entrepreneurial practices, which, when these become routines, will enable the growth of both the entrepreneur and the company.

### Limitations

As with most studies, this research is subject to several limitations, which are in turn associated with risks of bias. The main limitation is the likelihood that not all existing articles were included in the study, due to multiple causes, such as engine limitations and lack of included keywords in articles, even though the number of keywords and their derivations used was extensive to prevent this effect. This can improve the scope of the sample, but it can also add irrelevant articles and make the sample more challenging and less practical. Additionally, to account for this effect, entrepreneurs' well-being was analyzed without restrictions in the knowledge areas and without period restriction. The databases were also expanded to include more information.

## Author contributions

All three authors conceptualized and designed the review. GV-M structured and extracted some of the information and drafted the document. BH extracted additional information and added important details. JS-G reviewed the information and adjusted the protocol for data extraction. GV-M made the requested corrections and JS-G provided the final approval. All three authors have final approval of the published article and agree to be responsible for all aspects of the work to ensure that questions related to the accuracy or integrity of any part of the work are properly investigated and resolved.

### Conflict of interest statement

The authors declare that the research was conducted in the absence of any commercial or financial relationships that could be construed as a potential conflict of interest.

## Abbreviations

SSCI: Social Science Citation Index.
